# Mexiletine and False Positive Urine Drug Screen for Amphetamine: A Case Review

**DOI:** 10.1155/2021/7134394

**Published:** 2021-11-30

**Authors:** Sumit Sohal, Mina Sous, Gauri Pethe, Shanmugha V. Padmanabhan, Rajesh Akbari, Shahriar Dadkhah

**Affiliations:** ^1^Division of Cardiovascular Diseases, Department of Internal Medicine, Newark Beth Israel Medical Center, 201 Lyons Ave, Newark, NJ 60202, USA; ^2^Department of Internal Medicine, AMITA Health Saint Francis Hospital, 355 Ridge Avenue, Evanston, IL 60202, USA; ^3^Division of Cardiovascular Diseases, Department of Internal Medicine, AMITA Health Saint Francis Hospital, 355 Ridge Avenue, Evanston, IL 60202, USA

## Abstract

Advanced heart failure patients commonly suffer from ventricular arrhythmias which can be managed by antiarrhythmic drugs like mexiletine. These ventricular arrhythmias can be complicated by illicit drug use which alter outcomes and can potentially impact the patient-physician relationship through countertransference. However, mexiletine can lead to false positive urine drug screen testing for amphetamine, and these false-positive urine drug screen test results can affect the decision-making process. Health care providers should be aware of this fact and should either use confirmatory testing or look for confounding compounds in patients who deny using illicit substances and have a positive urine drug screen. Our patient is 64 years old who arrived at the emergency department after experiencing a shock by his intracardiac defibrillator. The patient tested positive for amphetamine on his urine drug screen and was later ruled out by confirmatory quantitative testing.

## 1. Introduction

Ventricular arrhythmia is common in patients who suffer from advanced heart failure, which can be managed by antiarrhythmic drugs such as mexiletine [1]. Mexiletine is a class Ib antiarrhythmic drug that is associated with false-positive urine drug screening (UDS) test for amphetamine, but negative on the confirmatory test [1, 2]. Here, we discuss a case of a 64-year-old male with heart failure with reduced ejection fraction on mexiletine who presented after experiencing a shock by his implantable cardiac defibrillator (ICD). His UDS for amphetamine was positive, even though he adamantly denied illicit drug use. This was deemed to be a false-positive result based on further confirmatory testing.

## 2. Case Presentation

The patient is a 64-year-old male with a medical history of hypertension, chronic kidney disease, atrial fibrillation, heart failure with reduced ejection fraction (nonischemic cardiomyopathy) with left ventricular ejection fraction of 35%, and ventricular tachycardia with ICD in place on amiodarone and mexiletine, who presented after experiencing a shock by his ICD twice on the day of admission. The patient complained of palpitations on the day of admission and denied having chest pain, shortness of breath, cough, or loss of consciousness. On physical exam, his blood pressure was 109/71 mmHg, heart rate was 100 beats per minute, respiratory rate was 18 breaths per minute, oxygen saturation was 96% on room air, and temperature was 97.8 F. He was alert and oriented, had clear breathing sounds, bilateral, no heart murmurs, and no leg swelling. EKG showed ventricular paced rhythm with underlying atrial fibrillation. Significant laboratory showed an increase in his creatinine to 1.7 mg/dl from a baseline of 1.2 mg/dl and a TSH of 16 uIU/mL and normal free thyroid hormone levels. The ICD interrogation showed multiple sustained monomorphic ventricular tachycardia episodes. During his hospital stay, his workup revealed positive UDS for amphetamine on 2 separate occasions (urine toxicology screen), but the patient adamantly denied using amphetamine or any other illicit drugs. He ultimately underwent confirmatory drug testing for amphetamine (quantitative urine amphetamines), which was negative. Ultimately, the patient underwent successful VT ablation and was discharged home.

## 3. Discussion

Ventricular arrhythmias occur frequently in patients with advanced heart failure and can be detected in up to 80% with congestive heart failure (CHF) [3]. The current treatment modalities include antiarrhythmic drugs, implantable cardioverter/defibrillator, and radiofrequency ablation [4]. Antiarrhythmic drugs suppress the tachycardia cycle and reduce the number of shocks fired by the ICD and can prolong the device's battery life [5]. Mexiletine is a class Ib antiarrhythmic drug that is used in combination with class III drugs such as amiodarone to achieve ventricular arrhythmia suppression as in our patient who was on both medications [6, 7]. It shares similar structural and electrophysiological properties with lidocaine and decreases ventricular automaticity [8]. It blocks fast sodium channels during phase 0 of the action potential and is significantly rate dependent, which makes it effective in ventricular tachyarrhythmias with minimal effect on normal conduction [2].

**Figure 1:**
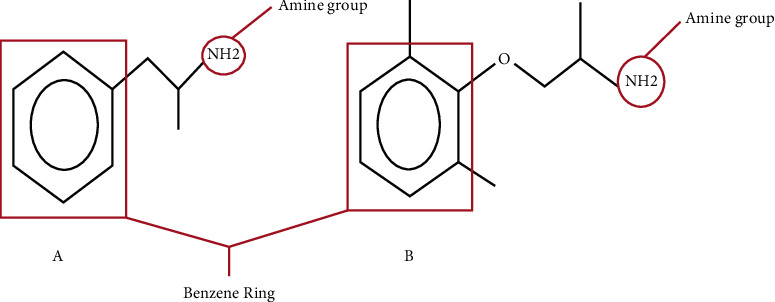
Similarity between amphetamine (A) and mexiletine (B) [1].

Mexiletine can lead to a positive UDS for amphetamine. The immunoassays currently used for UDS uses the technique of antibodies detecting the drug of interest or its metabolites. It is believed that there is cross-reactivity due to the structural similarity of both compounds, as both drugs have a benzene ring with a hydrocarbon tail that ends with an amine group (Figure 1) [1]. Other commonly prescribed medications also interfere with the UDS such as antihistamines, antipsychotics, and other drugs such as amantadine, benzophetamine, buproprion, carbidopa/levopdopa, chlorpromazine, desipramine, doxepin, ephedrine, fluoxetine, phenylephrine, ranitidine, selegiline, and trazodone [9]. It is important to recognize these drugs as causative agents that can cause false-positive UDS as these are frequently prescribed drugs. Quantitative tests can be used to identify the drug. These tests are confirmatory tests with some laboratories offering reflex quantitative testing after initial positive screening, and the test may take 1–4 days to result. Liquid chromatography with mass spectrometry (LC-MS) can provide high specificity and sensitivity in analyzing molecules' size and structure, as it converts the molecules to smaller fragments and segregates/identifies them based on the ionization state and charge/mass ratio to identify the compound of interest [10, 11].

Patients with advanced heart failure may be amenable for more invasive treatment options such as left ventricular-assisted devices or cardiac transplantation [12, 13]. Illicit drug use is an absolute contraindication to transplantation procedures. That is the reason for testing patients for drugs of abuse before undergoing such procedures. False-positive UDS can have significant implications and can potentially change management [14]. It is important for decision-making that healthcare providers realize that there are medications that can lead to false-positive UDS results and should look for potential confounding compounds or use the confirmatory tests such as LC-MS before making life-changing decisions or potentially excluding patients from the transplantation list [1, 14].

## 4. Conclusion

False-positive urine drug screening tests are common in clinical practice and can lead to serious implications in the decision-making process. Mexiletine which is a commonly prescribed antiarrhythmic drug can lead to false-positive UDS for amphetamines, thereby impacting the physician-patient relationship. Health care providers should be vigilant and aware of these false-positive tests and should seek confirmatory investigations such as quantitative testing before finalizing clinical management.

## Data Availability

All the relevant data are provided in the article.
